# The Dynamic Regulation of Daxx-Mediated Transcriptional Inhibition by SUMO and PML NBs

**DOI:** 10.3390/ijms26146703

**Published:** 2025-07-12

**Authors:** Jiatao Gao, Tingting Liu, Dongmei Yang, Qinhui Tuo

**Affiliations:** 1 Key Laboratory of Vascular Biology and Translational Medicine, Medical School, Hunan University of Chinese Medicine, Changsha 410208, China; jtg@stu.hnucm.edu.cn; 2 Basic Research Center of Chinese and Western Medicine on the Prevention and Treatment of Vascular Diseases, Changsha 410208, China; 20242085@stu.hnucm.edu.cn (T.L.); dongmeiy@hnucm.edu.cn (D.Y.); 3 Key Laboratory for Quality Evaluation of Bulk Herbs of Hunan Province, School of Pharmacy, Hunan University of Chinese Medicine, Changsha 410208, China

**Keywords:** Daxx, SUMO, SUMOylation, transcription factor, PML NBs

## Abstract

SUMOylation plays a crucial role in regulating gene expression by promoting interactions between transcription factors and corepressors. Daxx, a multifunctional scaffold protein, specifically recognizes and binds SUMOylated transcription factors through its SUMO-interacting motifs (SIMs), acting as a transcriptional corepressor. In this review, we systematically elucidate the structural basis of the interaction between Daxx and SUMO, revealing the synergistic mechanism by which Daxx SIM phosphorylation and SUMO acetylation dynamically regulate Daxx function. In promyelocytic leukemia nuclear bodies (PML NBs), phosphorylation of Daxx’s SIM enhances its binding to SUMOylated PML, leading to the sequestration and inactivation of Daxx within PML NBs. Conversely, SUMO acetylation disrupts the electrostatic interactions between SUMO and SIMs, prompting the release of Daxx from PML NBs and its translocation to the nucleoplasm, where it inhibits the activity of transcription factors such as ETS1, GR, and SMAD4. Daxx SIMs are common binding sites for the interaction between SUMOylated transcription factors and Daxx, and different SUMOylated transcription factors may compete to bind to Daxx, which cross-regulates cellular life activities. This mechanism highlights the dynamic regulation of Daxx subcellular localization and transcriptional repression by SUMO and PML NBs, providing valuable insights into understanding Daxx-mediated transcriptional repression.

## 1. Introduction

Gene expression regulation is a complex process involving intricate interactions among proteins, chromatin, histones, and chromatin-modifying enzymes. Daxx (death domain-associated protein), a multifunctional protein, plays a pivotal role in transcriptional regulation, histone modification, cell cycle control, and chromatin repair [1,2,3]. As one of the proteins interacting with SUMO (Small Ubiquitin-like Modifier), Daxx contributes significantly to transcriptional repression [4]. SUMO is a small ubiquitin-like protein modifier involved in the regulation of gene expression [5,6]. Human gene expression is regulated by over 2000 transcription factors [7,8]. Moreover, the majority of repression domain sequences in these regulators contain SUMOylation sites, concise interaction motifs for recruiting corepressors, or structured binding domains for recruiting additional repressive proteins [9]. With few exceptions, SUMOylation of transcription factors are typically associated with transcriptional repression, achieved by promoting interactions with corepressors such as Daxx or by recruiting histone deacetylases [10,11,12]. Daxx, functioning as a transcriptional corepressor, interacts with SUMO-modified transcription factors via its SUMO interaction motifs (SIMs), thereby facilitating the assembly of transcriptional repression complexes. Furthermore, Daxx is a constitutive component of promyelocytic leukemia nuclear bodies (PML NBs), in which it interacts with PML. Multiple studies have suggested that PML NBs act as “storage depots” for Daxx, modulating its activity as a transcriptional corepressor [13]. Specifically, the sequestration of Daxx within PML NBs restrict its availability for repressing transcription, whereas its release from these depots enables it to exert its co-repressive functions. This dual regulation underscores the critical role of PML NBs in modulating the dynamic activity of Daxx in transcriptional repression. SUMO, a significant component protein of PML NBs, not only participates in the formation of PML NBs but also regulates the release of Daxx from PML NBs. In this review, we elucidated the structural basis of the interaction between SUMO and Daxx, emphasizing their synergistic roles in transcriptional repression. Furthermore, we discussed a potential mechanism through which Daxx exerts its repressive effects: post-translational modifications (PTMs), particularly SUMOylation and acetylation, dynamically regulate Daxx activity within PML NBs to regulate transcriptional repression.

## 2. SUMO and SUMOylation

Proteins are the primary executors of biological activities, and their proper functioning ensures that these activities proceed efficiently. Post-translational modifications (PTMs) play crucial roles in maintaining protein function. To date, over 20 types of PTMs have been identified, including phosphorylation, acetylation, methylation, glycosylation, ubiquitination, and SUMOylation. Among these PTMs, SUMOylation is particularly important due to its roles in modulating protein subcellular localization and protein–protein interactions. In addition, SUMOylation is integral to various biological processes, including chromatin remodeling, DNA repair, transcription regulation, and cell cycle progression, underscoring Its critical role in maintaining cellular homeostasis [14].

### 2.1. The SUMO Family

SUMOs are a class of protein post-translational modifiers similar in structure to ubiquitin (Ub). To date, more than 6000 proteins, primarily nuclear proteins, have been identified as target proteins of SUMOylation [15]. SUMOylation is a reversible process in which the C-terminus of SUMO covalently binds to a lysine (Lys) residue on the target protein [16,17]. Unlike ubiquitination, SUMOylation does not promote protein degradation but instead stabilizes target proteins and modulates their functions [18,19].

**Figure 1:**
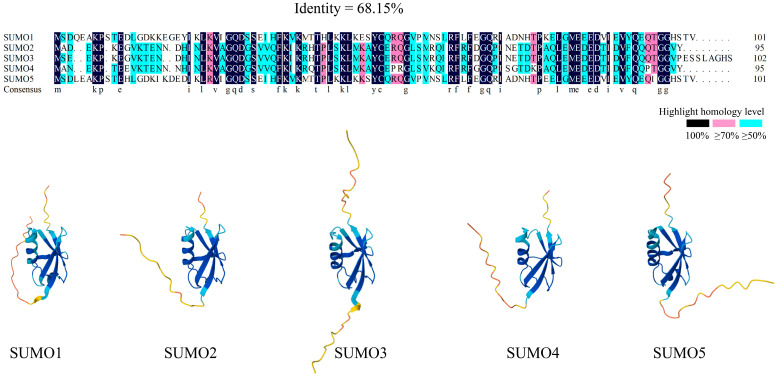
Homology alignments and three-dimensional structures of SUMO family. SUMO proteins exhibit high structural similarity in both their two-dimensional (amino acid sequence) and three-dimensional structures. The sequence similarity of SUMO1-SUMO5 in the homology comparison is 68.15%. Amino acids in the black sites show 100% similarity, those in pink sites exhibit >=70% similarity, and residues in blue sites demonstrate >=50% similarity (where letters denote amino acid residues in SUMO protein sequences). The blue region in SUMO three-dimensional structures denotes the conserved ubiquitin-like domain. Furthermore, significant sequence and structural homology exists among the SUMO paralogs themselves (SUMO1, SUMO2, SUMO3, SUMO4, and SUMO5).

In humans, five SUMO homologous genes have been identified: SUMO1, SUMO2, SUMO3, SUMO4, and SUMO5 [20]. Structurally, SUMOs share a high degree of similarity with ubiquitin, as depicted in Figure 1, where the blue region of the SUMO three-dimensional structure represents the ubiquitin-like domain. SUMO1, the first identified member of the SUMO family, primarily functions under normal physiological conditions, modifying proteins involved in routine biological activities [21]. SUMO2 and SUMO3 share 96% sequence homology and are predominantly activated during cellular stress [17,22,23,24]. These two isoforms often form poly-SUMO chains, which participate in stress response pathways. SUMO4 and SUMO5, the most recently identified members of the SUMO family, remain less well-studied. SUMO4 is encoded by the human TAB2 gene and resembles SUMO2/3 in both structure and function, with the 90th amino acid in SUMO4 being proline instead of glutamine, potentially affecting its function [25]. SUMO5, the latest addition to the SUMO family, is associated with the assembly and disassembly of promyelocytic leukemia nuclear bodies (PML-NBs) [20].

### 2.2. The SUMOylation Cascade

**Figure 2:**
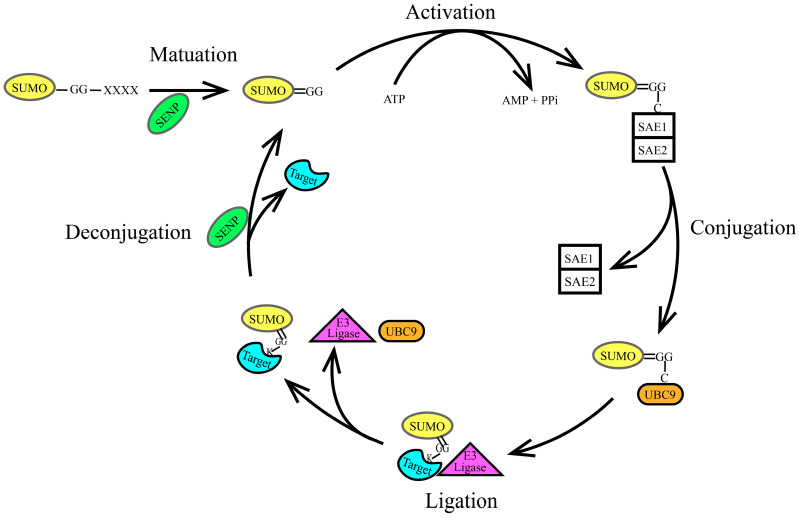
SUMOylation process. SUMOylation is a reversible process consisting of the following five steps: maturation, activation, conjugation, ligation, and de-modification, as illustrated in Figure. (1) Maturation: SUMO starts as an inactive precursor molecule. SENPs cleave the four amino acids at the C-terminus, exposing the terminal Gly-Gly (GG) motif, as shown in the figure, changing from SUMO-GG-XXXX to SUMO=GG. (2) Activation: The heterodimer SAE1-SAE2 (E1 activating enzyme) activates SUMO by linking the carboxyl group at the C-terminus of SUMO to a cysteine residue in the SAE1-SAE2 dimer through a thioester bond. This process requires ATP. (3) Conjugation: The activated SUMO protein is transferred to the conserved cysteine residue at position 93 of Ubc9 through an ester exchange reaction, forming an E2-SUMO thioester compound. (4) Ligation: The SUMO E3 ligase acts as an intermediary, linking the SUMO E2 complex to the target protein and facilitating the dissociation of SUMO from Ubc9. Finally, Ubc9 attaches the SUMO molecule to the lysine residue of the target protein via an iso-peptide bond. The precise positioning of the SUMO E2 complex by the SUMO E3 ligase reduces the distance between the thioester bond of the SUMO E2 complex and the substrate lysine residue, enhancing substrate specificity. (5) Deconjugation: SUMOylation is reversible; SENPs remove the SUMO protein from the lysine residue of the target protein, allowing the free SUMO protein to participate in another catalytic cycle.

**Figure 3:**
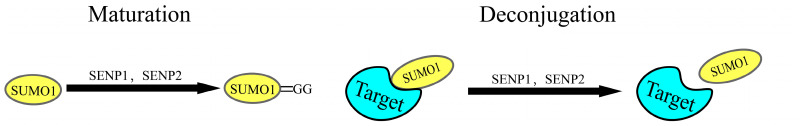

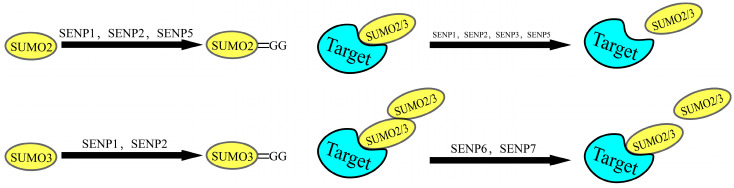
The Role of SENPs in SUMOylation. Sentrin-specific proteases (SENPs) play essential roles in SUMOylation. At the start of the SUMO cycle, SENPs catalyze the maturation of SUMO precursors by exposing the terminal Gly-Gly (GG) motif. Conversely, at the cycle’s termination, SENPs mediate deSUMOylation—cleaving the isopeptide bond to separate SUMO proteins from substrate targets.

SUMOylation is a reversible post-translational modification mediated by a conserved enzymatic cascade that shares mechanistic parallels with, but diverges functionally from, the ubiquitin conjugation pathway. This process involves the coordinated action of SUMO-specific enzymes, including E1 activating enzymes, E2 conjugating enzymes, E3 ligases, and sentrin-specific proteases (SENPs) [26]. The most studied SUMO-specific enzymes are the SENP (sentrin-specific protease) family, which includes SENP1, SENP2, SENP3, SENP5, SENP6, SENP7, and SENP8 [27,28]; however, SENP8 does not act on SUMO [29]. SUMOylation is a reversible process consisting of the following five steps, as illustrated in Figure 2: maturation, activation, conjugation, ligation, and de-modification. At the start of the SUMO cycle, SUMO exists as an inactive precursor that requires SENPs to cleave its C-terminal tail, exposing the terminal Gly-Gly (GG) motif. SENPs also play a crucial role in the dissociation of SUMO from the target protein. Different types of SENPs interact with various SUMO isoforms, as illustrated in Figure 3. SENP6 and SENP7 exhibit the activity to dissociate poly-SUMO2/3 chains [29]. The E1 activating enzyme is typically a dimer composed of SUMO-Activating Enzyme 1(SAE1) and SUMO-Activating Enzyme 2 (SAE2), with SUMO molecules often binding to SAE2; currently, the only reported E2 conjugating enzyme is Ubiquitin Conjugating Enzyme 9 (UBC9). In contrast, the diversity of E3 ligases depends on the specific substrates, enhancing the substrate specificity of the modification process [30,31,32].

## 3. Interaction Between Daxx and SUMO

### 3.1. Structure and Biological Characteristics of Daxx

**Figure 4:**
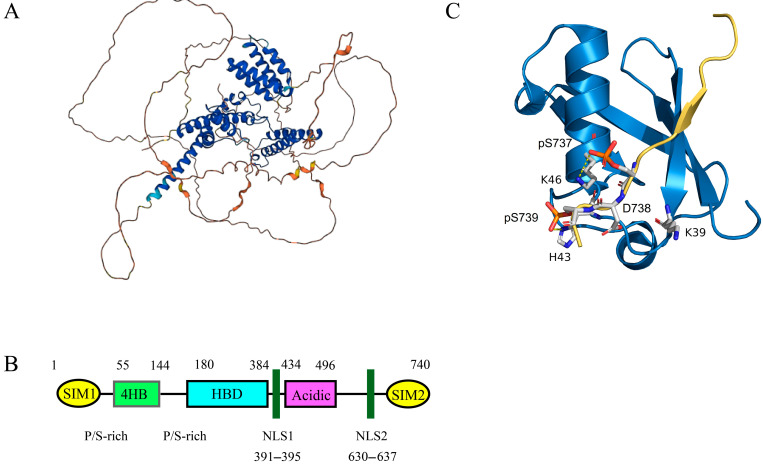
(**A**) The three-dimensional structure of Daxx. AlphaFold assigns per-residue confidence scores (pLDDT, 0-100) to its structural predictions, visualized here by color: regions in purple represent very high confidence (pLDDT > 90), blue denotes high confidence (70 <= pLDDT < 90), orange indicates low confidence (50 <= pLDDT < 70), and red signifies very low confidence (pLDDT < 50). Residues scoring below pLDDT 50 frequently correspond to intrinsically disordered segments that may lack stable tertiary structure in isolation. (**B**) Daxx includes the following components: (1) SIM: SUMO Interaction Motif; (2) HBD: Histone Binding Domain; (3) 4HB: Daxx Helical Bundle; (4) NLS: Nuclear Localization Signal. (**C**) The structure of the phosphorylated Daxx SIM2 bound to SUMO 1 (PDB ID 4WJP). In this figure, the blue area represents SUMO 1, and the yellow area represents Daxx SIM2. The three negatively charged amino acid residues on Daxx SIM2 (phospho-Ser737, Asp738, and phospho-Ser739) and the three positively charged amino acid residues on SUMO 1 (Lys39, His43, Lys46) enable the binding of Daxx SIM2 to SUMO 1 in a parallel direction due to positive-negative charge interactions.

Daxx (death domain-associated protein 6) was initially identified as a Fas-binding Protein and serves as a regulator of cell death involved in JNK-mediated apoptosis [33,34]. Daxx co-localizes with PML in PML-NB, which is also known as nuclear domain 10 (ND10) [33]. Daxx and its homologous genes are exclusively found in the animal kingdom and are widely distributed across most human tissues and organs, playing a crucial role in embryonic growth and development [35,36,37]. The Daxx gene is located on human chromosome 6 at the 6p21.3 region, which is part of the major histocompatibility complex (MHC) area. Daxx consists of two helical domains (the 4HB domain and the HBD domain), an acidic domain rich in acidic amino acids (Daxx acidic domain), and a domain rich in serine, proline, and threonine (S/P/T domain) [38]. Figure 4 illustrates the three-dimensional structure and two-dimensional unfolded structure of Daxx [39].

Daxx interacts with various proteins in both the cytoplasm and nucleus, functioning as a transcriptional corepressor or co-activator by interacting with multiple DNA-binding transcription factors, core histones, and chromatin-associated proteins, thereby regulating gene expression [40,41,42,43,44]. The structure of Daxx contains two SUMO-binding motifs (SIMs), located at its N-terminus (SIM1) and C-terminus (SIM2), which are essential for its interactions with various proteins and transcription factors [35,45]. As a chaperone for the histone variant H3.3, Daxx is also a significant chromatin regulatory factor that influences chromatin stability and maintains telomere length [46,47,48,49]. Daxx can also prevent the misfolding of β-amyloid and α-synuclein proteins and can restore the misfolded P53 protein to its native conformation [50].

### 3.2. Structural Basis of Daxx Binding to SUMO

Daxx is a multifunctional scaffold protein that interacts with various proteins, including SUMO proteins. The SUMO Interaction Motifs (SIMs) in Daxx are crucial for its binding with SUMO. Daxx contains two SIMs: one at its N-terminus (SIM1) and another at its C-terminus (SIM2). Both SIM1 and SIM2 have identical hydrophobic centers and contain phosphorylatable residues [51]. The two SIMs are highly conserved evolutionarily and are rich in glutamic acid (Glu) and aspartic acid (Asp) residues, with four consecutive leucine (Leu) or isoleucine (Ile) residues forming the hydrophobic center. The SIMs can insert into the groove on the surface of SUMO-1, which is formed by four basic residues: Lys37, Lys39, Lys46, and Arg54 [4,52]. This interaction is essential for the binding of Daxx to SUMO-1.

Although both SIM1 and SIM2 can bind to SUMO proteins in a parallel orientation, SIM1 has a binding affinity for SUMO1 and SUMO2 that is four times higher than that of SIM2 [51,53]. Interestingly, when serine residues (Ser-737 and Ser-739) in SIM2 are phosphorylated by casein kinase 2 (CK2), the affinity of SIM2 for SUMO1 increases approximately 30-fold [51]. Furthermore, phosphorylated SIM2 binds more tightly to SUMO1 than to SUMO2. Phosphorylation of SIMs increases the number of negatively charged residues flanking the hydrophobic core, enhancing the binding affinity between Daxx and SUMO through electrostatic interactions with positively charged residues in SUMO [54,55,56]. SIM1, also contains two evolutionarily conserved phosphorylation sites (Thr-4 and Ser-7), but it remains unclear whether phosphorylation at these sites enhances the interaction between SIM1 and SUMO. Additionally, there is a weak intramolecular interaction between the intrinsically disordered region (IDR) of Daxx (amino acids 1–56) and its four-helix bundle domain (4HB), which can interfere with the interaction between Daxx and SUMO, as well as with the binding of proteins like p53, MDM2, and RASSF1C to the 4HB [53].

Many proteins can bind to the C-terminus of Daxx (SIM2), including FAS [33], PAX3 [40,43], PML [57], GLUT4 [58,59,60,61], ETS1 [62], SREBP [63], HIV-1 protein P6 [64], and hantavirus nucleocapsid protein PUUV-N [65]. Notably, these proteins can be SUMO-modified or interact with the E2 conjugating enzyme Ubc9. Prof. Shih and colleagues have also proposed that Daxx acts as a SUMO reader in SUMO-dependent transcription and subnuclear compartmentalization regulation; various SUMOylated factors can compete for interaction with Daxx, leading to cross-regulation of cellular events [4].

### 3.3. SUMOylation of Daxx

Daxx contains 15 lysine residues that can be modified by SUMO, with lysine 630 (K630) and 631 (K631) identified as the primary SUMOylation sites. However, SUMOylation of Daxx does not enhance its binding affinity for SUMOylated transcription factors, nor does it facilitate the transport of Daxx from the cytoplasm to the nucleus [45,66]. This indicates that the nucleocytoplasmic transport of Daxx and its interaction with transcription factors are not mediated by Daxx’s own SUMOylation.

The interaction between SUMO proteins and Daxx occurs in two forms: one involves non-covalent binding through Daxx’s SUMO Interaction Motifs (SIMs), and the other involves covalent modification of Daxx’s lysine residues by SUMO proteins. Notably, regardless of whether the interaction is non-covalent or involves covalent modification, SUMO proteins interact non-covalently with Daxx’s SIMs [67]. These SUMO–SIM interactions are crucial for the function of Daxx, but the SUMOylation of Daxx itself does not seem to play a significant role.

Based on the above discussion, we can infer that Daxx recognizes SUMOylated factors primarily through its SIMs and interacts with SUMO-modified proteins. Furthermore, this interaction may be regulated by the phosphorylation of Daxx SIMs and competitive binding from other SUMOylated factors.

## 4. Regulation of Daxx-Mediated Transcriptional Repression by SUMO Within PML Nuclear Bodies

### 4.1. Structure of PML NBs

The eukaryotic cell nucleus contains several substructures known as nuclear bodies, including Cajal bodies, nuclear speckles, paraspeckles, and promyelocytic leukemia nuclear bodies (PML NBs) [68,69]. PML NBs, also referred to as PML oncogenic domains, nuclear domain 10 (ND10), Kremer bodies, or membrane-less organelles, are dynamic macromolecular complexes composed of a PML-protein core and various transient or permanent components, forming spherical and dense structures [70]. The main proteins present in PML NBs include PML protein, ATRX/Daxx, SUMO, Sp100, Sp110, HP1, and p53, among others [71].

PML protein serves as the primary organizing molecule of PML NBs, with different splice variants resulting in diverse C-terminal structures, leading to the emergence of several isoforms, namely PML I-VII. PML can be covalently modified by SUMO1 and SUMO2/3 proteins at lysine residues K65, K160, and K490 [72,73,74,75]. In addition to these SUMOylation sites, there is a SUMO Interaction Motif (SIM) at the C-terminus of PML, composed of four hydrophobic residues (Val-Val-Val-Ile) along with a cluster of serine and acidic residues [76,77,78]. Similarly to Daxx, the SIM on PML can be phosphorylated, which enhances its interaction with SUMO [79]. The interaction between the SIM on PML and SUMO plays a crucial role in the assembly of PML NBs [45,75,77,80].

### 4.2. Role of SUMO in the Assembly of PML NBs

**Figure 5:**
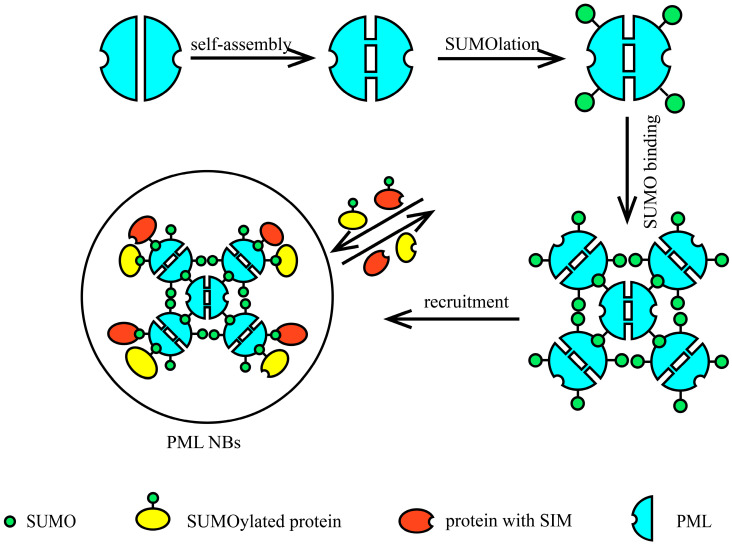
The process of PML NB assembly. The assembly of PML nuclear bodies (PML NBs) occurs through sequential steps: (1) PML monomers undergo self-assembly to form homodimers; (2) these dimers undergo SUMOylation at specific lysine residues; (3) multiple SUMOylated PML dimers oligomerize via interactions between their SUMO-interaction motifs (SIMs), forming higher-order PML multimers; (4) these multimers serve as scaffolds to recruit additional SUMOylated proteins and SIM-containing proteins, culminating in mature PML NBs.

The assembly of PML NBs begins with the aggregation of PML protein. PML self-aggregates through the oxidation of its cysteine residues by reactive oxygen species (ROS), forming disulfide bonds and engaging in non-covalent interactions via its RING finger, B-box, and coiled-coil (RBCC) domain. Studies indicate that specific PML isoforms may also participate in the initial steps of PML assembly. The second step involves the SUMOylation of PML protein. PML NBs are rich in the SUMO E2 conjugating enzyme UBC9, which mediates the SUMOylation of PML and other proteins within PML NBs [77]. Finally, PML recruits SUMO-modified proteins and proteins containing SIMs to the core structure composed by PML, utilizing its own SIM and SUMOylation [41,77,81,82,83,84]. These proteins transiently or permanently associate with PML to form dynamic macromolecular complexes known as PML NBs. In this way, PML drives liquid–liquid phase separation through SUMO–SIM interactions, ultimately completing the assembly of PML NBs. The assembly process of PML NBs is illustrated in Figure 5.

### 4.3. Dynamic Regulation of Daxx Localization Within PML NBs Through Post-Translational Modifications

PML NBs are involved in numerous critical cellular processes, including chromatin remodeling, transcription regulation, DNA damage response, tumor growth repression, apoptosis, antiviral responses, and the regulation of telomere length [85,86,87,88,89]. Three main hypotheses regarding the functions of PML NBs have emerged: (1) Protein storage warehouse: PML NBs serves as reservoirs for proteins; (2) Post-Translational Modification sites: It act as sites for post-translational modifications of proteins; (3) Transcription regulation and chromatin remodeling: PML NBs is implicated in transcription regulation and chromatin remodeling [90,91,92,93,94,95].

Nearly all proteins residing in PML NBs can undergo SUMO modification or possess SUMO Interaction Motifs (SIMs), enabling them to interact with SUMOylated PML [41,96,97]. The SUMOylation process is reversible, and non-covalent interactions between SUMO and SIMs can be dynamically regulated through post-translational modifications (PTMs), which can alter the charge of the interacting proteins and disrupt these interactions [98,99]. Consequently, the binding of PML to aggregated proteins in PML NBs is a dynamic and reversible process. Based on the dynamic binding mechanism between PML and its target proteins, PML can effectively function as a protein “warehouse” [55,100,101].

Through the regulation of PTM Networks, proteins within PML NBs can be released from or re-recruited to PML NBs in response to stress or other physiological stimuli [55]. Casein kinase 2 (CK2) is a ubiquitous kinase that mediates the phosphorylation of serine residues at positions 512, 513, 514, and 517 in the PML SIM [102]. The C-terminus of PML contains three identical CK2 phosphorylation sites, and phosphorylation at these three sites can generate a fourth identical phosphorylation site [103]. The CK2-mediated phosphorylation of PML SIM residues enhances the binding affinity between PML and SUMO proteins. Similarly, many proteins within PML NBs, such as Daxx and PIAS1, also possess SIMs that can undergo phosphorylation, which in turn regulates their non-covalent interactions with SUMO proteins [35,104,105,106,107].

According to the statements in the paragraph regarding the structural basis of Daxx binding to SUMO, the upregulation of CK2 promotes the phosphorylation of Daxx SIMs, facilitating the binding of Daxx with SUMOylated PML, thereby increasing the localization of nuclear Daxx within PML NBs [38,108]. In addition to phosphorylation, the interaction between Daxx SIM and SUMO is also influenced by acetylation of several key lysine residues within the SUMO SIM binding region [100,109]. Acetylation of SUMO1 at Lys37 and SUMO2 at Lys22 decreases the non-covalent interactions between Daxx and SUMOylated PML in PML NBs. The substitution of acetylated glutamine for SUMO1 at Lys37 inhibits the recruitment of Daxx to PML NBs. Moreover, compared to wild-type SUMO, acetylation-mimicking SUMO1/2 mutants exhibit significantly reduced binding to endogenous Daxx, indicating that SUMO acetylation interferes with Daxx recruitment to PML NBs by inhibiting SUMO–SIM interactions [100]. The binding of SUMO1 to phosphorylated SIMs of PML and Daxx can be altered by acetylation at Lys39, Lys46, and Lys37, with acetylation at Lys37 having a more pronounced effect on the interaction between phosphorylated Daxx SIM and SUMO1 [110]. Acetylation of lysine residues in SUMO could neutralize the electrostatic interactions between the SIM and SUMO proteins, leading to a decrease in Daxx within PML [100,110].

Based on the above experimental results and the assembly mechanisms of PML NBs, we can propose a possible mechanism by which SUMO’s role in the storage and release of Daxx, thereby modulating Daxx-mediated transcriptional repression. When CK2 levels are elevated in PML NBs, phosphorylation occurs on the SIMs of PML and Daxx, enhancing their binding capacity with SUMO proteins. This leads to an increased quantity of SUMOylated PML, effectively sequestering Daxx and other PML NB component proteins within PML NBs. The sequestration of Daxx within PML NBs could not exert the function of transcriptional repression because Its activity is inhibited. Conversely, under altered physiological conditions or in response to specific stimuli, SUMO protein acetylation disrupts the PML–SUMO–Daxx complex. This disruption results in the release of Daxx from PML NBs, allowing it to exert its transcriptional repression function and thereby enhancing transcriptional repression.

## 5. The Interaction of Daxx with Specific Transcription Factors in a SUMO-Depend Manner

SUMO proteins play a pivotal role not only in the assembly of PML and the storage and release of Daxx within PML nuclear bodies but also as a “bridge” that mediates the interaction between Daxx and some transcription factors. This interaction enhances the capacity of Daxx to function as a transcriptional corepressor. As illustrated in Table 1, Daxx interacts with various transcription factors, thereby inhibiting their transcriptional activity.

### 5.1. Daxx Inhibits the Transcriptional Activity of ETS1 in a SUMO-Dependent Manner

Daxx inhibits the transcriptional activity of ETS1 (E26 Transformation-Specific Transcription Factor 1) in a SUMO-dependent manner. ETS1, a member of the ETS transcription factor family, is widely distributed across various tissues and organs [128,129]. It plays significant roles in lymphoid tissue development, heme synthesis, angiogenesis, apoptosis, and anti-apoptosis [130,131,132,133,134,135]. Daxx interacts with ETS1 in the nucleus, and this interaction suppresses ETS1’s transcriptional activity, leading to decreased expression of its downstream targets, MMP1 and BCL2 [62]. Notably, Daxx does not bind directly to ETS1. The 15th lysine residue on the disordered N-terminus of ETS1 serves as a SUMO-1 modification site. After SUMO-1 covalently attaches to ETS1 via an isopeptide bond, Daxx’s SIM-C recognizes SUMO-1, bridging ETS1 in a “bead-on-a-string” manner. Subsequently, Daxx recruits HDAC and other factors to form a transcriptional repression complex, inhibiting ETS1’s transcription of associated genes such as MMP1 and BCL2 [53,62].

### 5.2. Daxx Inhibits the Transcriptional Activity of GR in a SUMO-Dependent Manner

The glucocorticoid receptor (GR), a steroid hormone produced by the adrenal cortex, is involved in development, anti-inflammatory responses, and metabolic regulation [136,137,138]. Daxx functions as a transcriptional corepressor for GR by binding to it and inhibiting the transcription of GR-regulated proteins [45]. The C-terminal region of Daxx (SIM2) provides the structural basis for this function. When PML is overexpressed, the transcriptional repression of GR by Daxx is diminished; however, PML neither alters the distribution of GR nor does it bind directly to GR [113]. Instead, PML sequesters more Daxx in the N10 domain, preventing its activity. Consistent with this hypothesis, the interaction between GR and Daxx is also mediated by SUMO proteins as a “bridge”. Treatment with As_2_O_3_ enhances the aggregation of PML and the recruitment of Daxx to PML NBs. Following As_2_O_3_ treatment, the movement of Daxx to the promoter regions of GR-regulated genes decreases, indicating a competitive recruitment effect between SUMOylated PML and SUMOylated GR for Daxx [45].

### 5.3. Regulation of Pax3 Transcriptional Activity Through Daxx Release from PML NBs

Paired Box 3 (Pax3) is a transcription factor critical for embryonic development and cell proliferation, particularly in the neural crest and muscle progenitor cells. Mutations in Pax3 are linked to Waardenburg syndrome and other developmental disorders [139]. Daxx has been shown to interact with Pax3, inhibiting approximately 80% of Pax3 transcriptional activity, a process dependent on the structure and function of the C-terminus of Daxx [40]. Overexpression of PML reduces the repressive effect of Daxx on Pax3. Increased PML leads to enhanced recruitment of Daxx to PML nuclear bodies (NBs), sequestering Daxx and preventing its role as a transcriptional corepressor. Similarly, treatment with IFNα and As_2_O_3_ causes Daxx to relocate within PML NBs, alleviating its inhibition of Pax3 transcription. Notably, overexpression of SUMO-binding defective PML does not diminish Daxx’s repression of Pax3, and there is currently no direct evidence that SUMOylation of Pax3 is required for the inhibitory effect of Daxx [43]. Moreover, there is also no direct evidence to suggest that Pax3 undergoes SUMOylation. However, other members of the Pax gene family, such as Pax5, Pax6, and Pax7, have been shown to be SUMOylated [140,141,142]. Interestingly, Daxx functions as a transcriptional coactivator for Pax5, enhancing its transcriptional activity within B cells [143].

### 5.4. Daxx Inhibits the Transcriptional Activity of SMAD4 in a SUMO-Dependent Manner

SMAD4 is a member of the SMAD protein family, primarily involved in regulating the TGF-β signaling pathway [144]. Daxx acts as a transcriptional corepressor for SMAD4, and deletion of the C-terminus of Daxx abolishes its inhibitory effect on SMAD4 transcription. Daxx does not inhibit SMAD4’s transcriptional activity by altering its nuclear translocation. The SUMOylation of SMAD4 at Lys159 is critical for Daxx-induced transcriptional repression; a mutation at this site results in the loss of Daxx’s repressive effect [115].

### 5.5. Involvement of SUMOylation in Daxx-Mediated Inhibition of AR Transcriptional Activity

Daxx interacts with the androgen receptor (AR) to inhibit AR-mediated transcriptional activation, including the repression of the AR-regulated gene PSA [116,145,146]. Overexpression of Daxx does not affect the overall expression of AR or its nuclear localization. Instead, Daxx binds to the N-terminal and DNA-binding domains of AR, preventing or competing with AR’s binding to target DNA. Mutations at the SUMOylation sites K386 and K520 of AR reduce the interaction between Daxx and AR by 30%. SUMOylated AR can recruit more Daxx, further enhancing Daxx’s inhibitory effect on AR’s transcriptional activity, although Daxx’s binding to AR is not entirely dependent on AR SUMOylation [116].

### 5.6. Daxx’s Inhibition of AIRE Transcriptional Activity Via Its C-Terminal Domain

AIRE is an unconventional transcription factor that promotes the expression of numerous genes in medullary thymic epithelial cells, facilitating the deletion or diversion of self-reactive T cells [147,148,149]. The interaction between AIRE’s HSR/CARD domain and Daxx’s Ser/Pro-rich C-terminal domain inhibits AIRE’s transcriptional activity. While the formation of AIRE homodimers is essential for its transcriptional activation, Daxx does not inhibit this process directly; rather, it recruits HDACs to compact chromatin, thereby suppressing AIRE’s transcription. Notably, GST-pulldown assays indicate that Daxx does not directly bind to the GST-AIRE fusion protein (aa 1–161) and requires additional bridging proteins to form the Daxx–AIRE complex [118].

### 5.7. The Inhibition of SNAI2 Transcriptional Activity by Daxx May Require the Participation of SUMO

Daxx inhibits the transcriptional activity of SNAI2 (also known as Slug), which is encoded by the SNAI2 gene and is a member of the Snail family of zinc-finger transcription factors. SNAI2 is highly conserved across vertebrate species and is considered a prototypical transcription factor involved in epithelial-to-mesenchymal transition (EMT) [150,151]. Daxx interacts with SNAI2 in the nucleus, and this interaction can suppress SNAI2’s transcriptional activity. However, the half-life of the Slug protein remains unchanged in the presence or absence of Daxx, suggesting that Daxx binding may not affect Slug protein stability. It is also possible that the interaction between Daxx and Slug requires another unidentified protein [119]. Interestingly, SUMO can inhibit Slug’s transcriptional activity by recruiting additional HDAC1, leading to decreased expression of downstream Slug target genes [152]. Daxx, as a transcriptional corepressor, primarily functions by recruiting HDAC1, and could bind to SUMO. Based on the evidence that Daxx interacts with some transcription factors requiring SUMO as a bridge, we speculate that SUMO may be the bridging protein of Daxx interacting with SNAI2.

## 6. Potential Pathway of Daxx-Mediated Transcriptional Repression

In addition to inhibiting the transcriptional activity of SNAI2, Daxx also interacts with other transcription factors such as MR, TCF4, STAT3, MENIN, and NF-kappaB, and can suppress their transcriptional activity [122,124,126,153,154,155,156,157]. These transcription factors can also undergo SUMO modification, which alters their transcriptional activity [125,158,159,160,161,162,163,164]. For instance, Fas-associated factor 1 (FAF1) inhibits MR’s transcriptional activity in a SUMO-dependent manner [165]. Additionally, Daxx, upon binding to transcription factors, recruits various regulatory enzymes such as HDACs, DNMT1, and DMAP1, thereby exerting transcriptional repression or activation functions [62,157,166,167,168].

**Figure 6:**
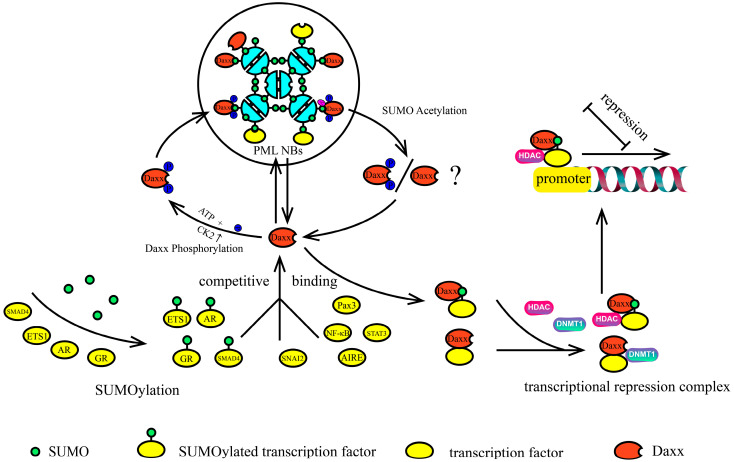
Possible mechanisms of Daxx-mediated transcriptional repression “Daxx-mediated repression operates through a phosphorylation-acetylation toggle: (1) CK2-mediated SIM phosphorylation enhances Daxx retention in PML NBs through stabilized PML–SUMO–Daxx complexes; (2) SUMO acetylation triggers Daxx release into the nucleoplasm; (3) Daxx binds SUMOylated transcription factors and recruits HDACs, DNMT1, and other epigenetic modifiers to form a transcriptional repression complex. Furthermore, competitive binding of different SUMOylated transcription factors to Daxx further modulates this process. “ATP+” indicates consumption of energy; “CK2 ↑” denotes elevated CK2 activity; “Ⓟ” represents a phosphoryl group; “Ⓐ” signifies an acetyl group.

Based on the above discussion, we can speculate a possible mechanism by which Daxx inhibits the transcriptional activity of transcription factors (as shown in Figure 6): When CK2 levels are elevated in PML nuclear bodies (PML NBs), phosphorylation occurs at the SUMO interaction motif (SIM) on PML and Daxx. This leads to an increased formation of the PML–SUMO–Daxx complex, sequestering Daxx within PML NBs and preventing its action. When the SUMO proteins within the PML-SUMO-Daxx complex undergo acetylation, the interaction between the components of the complex is disrupted, releasing Daxx from PML NBs. Once in the nucleoplasm, Daxx recognizes SUMOylated transcription factors through its SIM and binds to them, forming a Daxx–SUMO–transcription factor complex. Subsequently, Daxx recruits HDACs, DNMT1, DMAP1, and other regulatory enzymes to form a transcriptional repression complex that inhibits transcription. Additionally, various SUMOylated transcription factors or proteins may compete for binding with Daxx, which could depend on the priority of cellular events and the binding affinity of different SUMOylated molecules to Daxx.

## 7. Subcellular Localization of Daxx and Disease Implications

### 7.1. Subcellular Localization and Functions of Daxx

**Figure 7:**
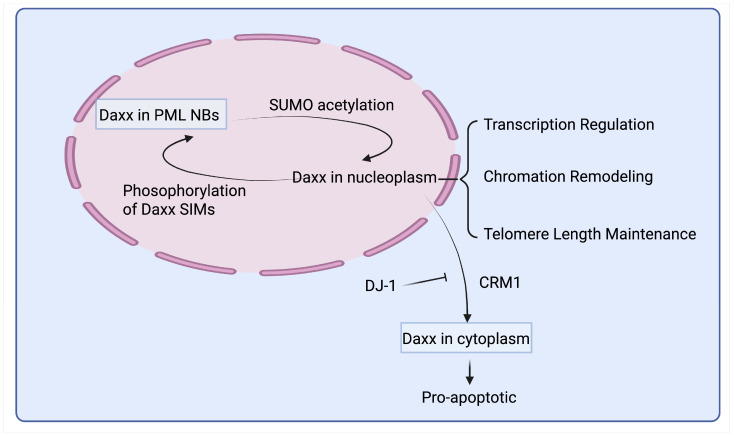
Subcellular distribution and function of Daxx. Daxx exhibits dynamic subcellular distribution, shuttling between the nucleus and cytoplasm. Within the nucleus, Daxx is largely stored in PML NBs. Phosphorylation of Daxx SIMs promotes the translocation of nucleoplasmic Daxx into PML NBs, while SUMO acetylation in PML NBs releases Daxx into the nucleoplasm. CRM1 mediates the nuclear export of Daxx to the cytoplasm, and DJ-1 inhibits Daxx’s nuclear export.

Daxx exhibits a dynamic subcellular distribution, shuttling between the nucleus and cytoplasm, as described in Figure 7, which is crucial for its diverse functions. In most cell types, Daxx is predominantly found within the nucleus. A significant portion of nuclear Daxx is concentrated in PML-NBs [39,169,170,171]. These dynamic, subnuclear structures are involved in a multitude of cellular processes, including transcriptional regulation, apoptosis, DNA repair, and antiviral responses. The SUMO-specific protease SENP1 promotes the release of Daxx from PML-NBs [172]. Beyond PML-NBs, Daxx is also distributed throughout the nucleoplasm and directly associates with chromatin. This localization is consistent with its established roles as a transcriptional coregulator and a histone chaperone involved in chromatin remodeling.

Although primarily nuclear, Daxx can also reside in the cytoplasm [170,171]. Cytoplasmic Daxx is particularly implicated in apoptotic signaling pathways, for example, through its interaction with the Fas receptor and ASK1 [33]. Cellular stress conditions can trigger the relocalization of Daxx from the nucleus to the cytoplasm, allowing it to engage these cytoplasmic pathways. The nuclear export of Daxx is mediated by the exportin chromosome region maintenance 1 (CRM1), while Parkinson’s disease protein 7 (PARK7, DJ-1) has been shown to inhibit Daxx’s export to the cytoplasm, thereby protecting cells from Daxx/ASK1-induced cell death [173,174,175].

### 7.2. Pathological Consequences of Aberrant Daxx Localization

#### 7.2.1. Enhanced Nuclear Export of Daxx May Exacerbate Parkinson’s Disease (PD)

Parkinson’s disease (PD) is a progressive neurodegenerative disorder primarily characterized by the loss of dopaminergic neurons in the substantia nigra. Mutations in the PARK7 gene, which encodes the protein DJ-1, are linked to autosomal recessive early-onset PD [176]. DJ-1 is recognized as a multifunctional protein with significant roles in protecting cells against oxidative stress [177]. Daxx can interact with DJ-1, and DJ-1 can inhibit the output of Daxx to the cytoplasm, thereby protecting cells from ASK1-induced cell death [174]. Oxidative stress and ASK1-mediated apoptosis are the key factors in the pathogenesis of pd. Therefore, the interaction between DJ-1 and Daxx may be an important neuroprotective mechanism. In PD cases, due to mutations or other factors, the function of DJ-1 is impaired, and the cytoplasmic transport of Daxx is disordered. This may lead to an increase in the cytoplasmic localization of Daxx and an enhanced activation of the ASK1 pathway, resulting in an increase in neuronal cell apoptosis [175,178]. Studies in Drosophila melanogaster models of PD have shown that mutants lacking DJ-1β (the fly ortholog of human DJ-1) exhibit increased neuronal death when subjected to oxidative stress [179]. While this particular study does not directly implicate Daxx, the established interaction between human DJ-1 and Daxx 2 suggests a potentially conserved pathway where DJ-1 exerts its neuroprotective effects, at least in part, by controlling Daxx’s pro-apoptotic potential.

#### 7.2.2. The Abnormal Release of Daxx in PML NBs Exacerbates Acute Promyelocytic Leukemia (APL)

Acute promyelocytic leukemia (APL) is a subtype of acute myeloid leukemia (AML). APL is driven by the t (15;17) (q22; q21) chromosomal translocation, which leads to the fusion of the promyelocytic leukemia (PML) gene with the retinoic acid receptor alpha (RARA) gene [180]. The resulting PML-RARA fusion protein disrupts normal RARA signaling [181]. The PML-RARA fusion protein can bind to retinoic acid response elements of target genes and recruit co-repressors such as DNA methyltransferases and histone deacetylases, and sequester retinoic X receptor and the wild-type PML protein, which finally leads to suppression of genes necessary for granulocytic differentiation [182]. APL causes structural disruption of PML NBs, leading to the abnormal release of Daxx from PML NBs. Daxx relocates to the nucleoplasm and heterochromatin [183], which may further exacerbate APL. Activation of PML in lymphocytes impairs the pro-apoptotic function of Daxx [184,185], which allows leukemic cells to escape Daxx-mediated programmed cell death (apoptosis). Furthermore, the PML-RARα fusion protein can recruit Daxx to nuclear microspeckled sites independently of PML NBs, resulting in the loss of Daxx’s pro-apoptotic activity [75]. On the other hand, Daxx acts as a transcriptional co-repressor for CCAAT/enhancer-binding Protein beta (C/EBPβ), potentially inhibiting normal myeloid differentiation of leukocytes [186]. C/EBPβ is a major PML-RARα-responsive gene in APL cells. Its expression is dramatically upregulated upon all-trans-retinoic acid (ATRA) treatment, and its transcriptional activity is essential for ATRA-induced APL differentiation [187]. During normal leukocyte differentiation, the formation of the Daxx-C/EBPβ complex decreases. Critically, both ATRA and arsenic trioxide treatment reduce the fraction of C/EBPβ associated with Daxx. This suggests that relief from Daxx-dependent repression of C/EBPβ is a crucial molecular event enabling APL cell differentiation [186]. In APL cells, both all-trans-retinoic acid (ATRA) and arsenic trioxide promote the reassembly of PML NBs. This leads to the re-recruitment of nucleoplasmic Daxx back into PML NBs [188], which may provide a new insight for the treatment of APL.

#### 7.2.3. SUMO2/3-Mediated Increase in Nuclear DAXX Aggravates Gastric Cancer (GC)

Gastric cancer (GC) ranks as the fifth most common malignancy and the fourth leading cause of cancer-related death globally [189,190]. Clinical studies reveal that Daxx expression is elevated in GC tissues, and tumor tissues exhibit a significantly higher nuclear/cytoplasmic ratio (NCR) of Daxx [191]. Critically, cytoplasmic Daxx and nuclear Daxx exert opposing effects on clinical features and survival outcomes in GC patients: high cytoplasmic Daxx expression correlates with lymph node metastasis, Lauren classification, and better overall survival. High nuclear Daxx expression is associated with higher recurrence rates and poorer survival. Experimental validation using nuclear/cytoplasmic fractionation assays in GC cells demonstrated that Daxx overexpression primarily increases cytoplasmic Daxx expression; silencing Daxx markedly reduces nuclear Daxx expression. Daxx overexpression significantly inhibits GC cell proliferation while promoting apoptosis, migration, and invasion. Knocking down Daxx robustly suppresses proliferation, anti-apoptotic effects, migration, and invasion in GC cells. These findings collectively indicate that elevated nuclear Daxx may be a key driver of aggravated malignancy in GC. The abnormal increase in SUMO2/3 may be the cause of the elevated nuclear Daxx in GC cells. Overall, Daxx expression decreased after knocking down SUMO-2/3 expression. However, when Daxx overexpression and SUMO-2/3 knockout coexist, the expression of Daxx in the cytoplasm and nucleus shows a decreasing trend, with a significant decrease in the expression in the nucleus. This suggests that SUMO2/3 may promote the increase in nuclear Daxx, but the experimental data cannot fully demonstrate that SUMO2/3 elevates nuclear Daxx by enhancing Daxx SUMOylation level. To conclusively establish this mechanism, an experiment of Daxx SUMOylation site mutation must be added [192].

## 8. Summery and Future Perspectives

Here, we describe the potential mechanisms of Daxx-mediated transcriptional repression, but several questions remain that require further research to determine. The interaction between the hydrophobic core of the Daxx SIM and the hydrophobic groove of SUMO proteins forms the basis for the binding of Daxx to SUMO proteins [51,52]. Phosphorylation of Daxx and PML SIM enhances their binding affinity to SUMO proteins by strengthening the electrostatic interactions between positive and negative charges [35,104,107]. Acetylation of lysine residues in SUMO could neutralize the electrostatic interactions between the SIM and SUMO proteins [100,110]. The mechanism of PTM that regulates the release of endogenous Daxx from PML NBs and decides whether to apply other component proteins within PML NBs, such as Sp100, HP1, and p53, warrants further investigation. Based on the increase in PML, it can compete with SUMOylated transcription factors to bind Daxx, thus reducing the inhibition of Daxx on transcription activity of transcription factors [43,45]. We can assume that various SUMOylated factors compete with each other to bind Daxx, leading to the crossover and dynamic regulation of cell life activities. Which SUMOylated factor can obtain the competitive advantage of binding with Daxx, whether it depends on the SUMO degree of SUMOylated factor itself or the main physiological activities of cells at the present stage?

Daxx, beyond its established role as a transcriptional repressor, has also been identified as a transcriptional coactivator [143,193]. The dual functionality of Daxx as both a corepressor and coactivator may be attributable to the distinct enzymes it recruits in each context. In its role as a corepressor, Daxx typically interacts with transcriptional inhibitory enzymes, including histone deacetylases (HDACs), DNA methyltransferase 1 (DNMT1), and DMAP1, facilitating changes in chromatin structure that suppress gene expression [62,157,166,167,168]. Conversely, when functioning as a co-activator, it remains to be elucidated whether Daxx engages with RNA polymerase, histone acetyltransferases (HATs), and methyltransferases. Following Daxx interaction with transcription factors, the mechanisms underlying the inactivation of the transcriptional repression complex warrant further investigation. Specifically, it is crucial to determine whether the components simply dissociate to regain their individual functions or whether they undergo degradation Via the ubiquitin-proteasome system (UPS). If it is the former, whether the action and mechanism of SUMO acetylation destruction of PML–SUMO–Daxx complex is also applicable to Daxx–SUMO–transcription factor complex is worth further consideration and research.

Undoubtedly, SUMO and PML NBs play a crucial role in Daxx-mediated transcriptional repression. As a transcriptional corepressor, the storage and release of Daxx in PML NBs require the involvement of SUMO proteins. It has also been demonstrated that the binding of Daxx to certain transcription factors necessitates SUMO proteins as scaffold proteins, with Daxx SIM playing a significant role in this process. Post-translational modifications (PTMs) drive the liquid-phase separation of Daxx within PML NBs by altering SUMO-SIM interactions, thereby dynamically regulating Daxx-mediated transcriptional repression. The dynamic regulation of Daxx translocation from PML nuclear bodies (PML NBs) to the nucleoplasm by SUMO ensures the proper and orderly functioning of the Daxx-mediated transcriptional regulatory network. Furthermore, the association of altered Daxx subcellular localization with diseases like PD, APL, and GC underscores the functional complexity of Daxx. Future research into Daxx subcellular localization and its functions within distinct subcellular compartments will provide new avenues for treating Daxx-related diseases, such as cancer.

## Figures and Tables

**Table 1:** Daxx interacting transcription factor and inhibits their transcriptional activity.

Daxx Interacting Transcription Factor	Whether It Is SUMOylated or Not	SUMOylation Site	Effect	References
ETS1	Yes	Lys15, Lys227	transcriptional repression	[62,111]
GR	Yes	Lys277, Lys293	transcriptional repression	[112,113]
Pax3	unclear		transcriptional repression	[43]
SMAD4	Yes	Lys159, Lys113	transcriptional repression	[114,115]
AR	Yes	Lys388, Lys521	transcriptional repression	[116,117]
AIRE	unclear		transcriptional repression	[118]
SNAI2 (Slug)	yes	Lys192	transcriptional repression	[119,120]
TCF7L2 (TCF4)	Yes	Lys297	transcriptional repression	[121,122]
STAT3	Yes	Lys451	transcriptional repression	[123,124]
MENIN	yes	Lys591	transcriptional repression	[125,126]
RELB (NF-kappaB)	Yes	unclear	transcriptional repression	[127]

This table lists several transcription factors that interact with Daxx. Among these, the binding of Daxx to SMAD4, ETS1, AR, and GR has been demonstrated to require SUMO as a bridging protein. Whether SUMO serves as a bridge for the interaction be-tween Daxx and other listed transcription factors remains unclear. However, the vast majority of these transcription factors can undergo SUMOylation.

## Data Availability

Not applicable.

## References

[1] Pinto L.M., Pailas A., Bondarchenko M., Sharma A.B., Neumann K., Rizzo A.J., Jeanty C., Nicot N., Racca C., Graham M.K. (2024). DAXX promotes centromeric stability independently of ATRX by preventing the accumulation of R-loop-induced DNA double-stranded breaks. Nucleic Acids Res..

[2] Groh S., Milton A.V., Marinelli L.K., Sickinger C.V., Russo A., Bollig H., de Almeida G.P., Schmidt A., Forné I., Imhof A. (2021). Morc3 silences endogenous retroviruses by enabling Daxx-mediated histone H3.3 incorporation. Nat. Commun..

[3] Puto L.A., Brognard J., Hunter T. (2015). Transcriptional Repressor DAXX Promotes Prostate Cancer Tumorigenicity via Suppression of Autophagy. J. Biol. Chem..

[4] Shih H.M., Chang C.C., Kuo H.Y., Lin D.Y. (2007). Daxx mediates SUMO-dependent transcriptional control and subnuclear compartmentalization. Biochem. Soc. Trans..

[5] Flotho A., Melchior F. (2013). Sumoylation: A regulatory protein modification in health and disease. Annu. Rev. Biochem..

[6] Chymkowitch P., Nguea P.A., Enserink J.M. (2015). SUMO-regulated transcription: Challenging the dogma. Bioessays.

[7] Lambert S.A., Jolma A., Campitelli L.F., Das P.K., Yin Y., Albu M., Chen X., Taipale J., Hughes T.R., Weirauch M.T. (2018). The Human Transcription Factors. Cell.

[8] Medvedeva Y.A., Lennartsson A., Ehsani R., Kulakovskiy I.V., Vorontsov I.E., Panahandeh P., Khimulya G., Kasukawa T., Drablos F. (2015). EpiFactors: A comprehensive database of human epigenetic factors and complexes. Database.

[9] DelRosso N., Tycko J., Suzuki P., Andrews C., Aradhana, Mukund A., Liongson I., Ludwig C., Spees K., Fordyce P. (2023). Large-scale mapping and mutagenesis of human transcriptional effector domains. Nature.

[10] Gill G. (2005). Something about SUMO inhibits transcription. Curr. Opin. Genet. Dev..

[11] Ouyang J., Gill G. (2009). SUMO engages multiple corepressors to regulate chromatin structure and transcription. Epigenetics.

[12] Rosonina E., Akhter A., Dou Y., Babu J., Sri T.V.S. (2017). Regulation of transcription factors by sumoylation. Transcription.

[13] Lindsay C.R., Morozov V.M., Ishov A.M. (2008). PML NBs (ND_10_) and Daxx: From nuclear structure to protein function. Front. Biosci..

[14] Sahin U., de The H., Lallemand-Breitenbach V. (2022). Sumoylation in Physiology, Pathology and Therapy. Cells.

[15] Sheng Z., Wang X., Ma Y., Zhang D., Yang Y., Zhang P., Zhu H., Xu N., Liang S. (2019). MS-based strategies for identification of protein SUMOylation modification. Electrophoresis.

[16] Talamillo A., Barroso-Gomila O., Giordano I., Ajuria L., Grillo M., Mayor U., Barrio R. (2020). The role of SUMOylation during development. Biochem. Soc. Trans..

[17] Yang Y., He Y., Wang X., Liang Z., He G., Zhang P., Zhu H., Xu N., Liang S. (2017). Protein SUMOylation modification and its associations with disease. Open Biol..

[18] Han Z.J., Feng Y.H., Gu B.H., Li Y.M., Chen H. (2018). The post-translational modification, SUMOylation, and cancer (Review). Int. J. Oncol..

[19] Hu M.M., Yang Q., Xie X.Q., Liao C.Y., Lin H., Liu T.T., Yin L., Shu H.B. (2016). Sumoylation Promotes the Stability of the DNA Sensor cGAS and the Adaptor STING to Regulate the Kinetics of Response to DNA Virus. Immunity.

[20] Liang Y.C., Lee C.C., Yao Y.L., Lai C.C., Schmitz M.L., Yang W.M. (2016). SUMO5, a Novel Poly-SUMO Isoform, Regulates PML Nuclear Bodies. Sci. Rep..

[21] Hay R.T. (2005). SUMO: A history of modification. Mol. Cell.

[22] Bernstock J.D., Yang W., Ye D.G., Shen Y., Pluchino S., Lee Y.J., Hallenbeck J.M., Paschen W. (2018). SUMOylation in brain ischemia: Patterns, targets, and translational implications. J. Cereb. Blood Flow Metab..

[23] Enserink J.M. (2015). Sumo and the cellular stress response. Cell Div..

[24] Niskanen E.A., Palvimo J.J. (2017). Chromatin SUMOylation in heat stress: To protect, pause and organise? SUMO stress response on chromatin. Bioessays.

[25] Zhang J., Chen Z., Zhou Z., Yang P., Wang C.Y. (2017). Sumoylation Modulates the Susceptibility to Type 1 Diabetes. Adv. Exp. Med. Biol..

[26] Chang H.M., Yeh E.T.H. (2020). SUMO: From Bench to Bedside. Physiol. Rev..

[27] Hickey C.M., Wilson N.R., Hochstrasser M. (2012). Function and regulation of SUMO proteases. Nat. Rev. Mol. Cell Biol..

[28] Bialik P., Wozniak K. (2017). SUMO proteases as potential targets for cancer therapy. Postepy Hig. Med. Dosw. (Online).

[29] Kunz K., Piller T., Muller S. (2018). SUMO-specific proteases and isopeptidases of the SENP family at a glance. J. Cell Sci..

[30] Varejao N., Lascorz J., Li Y., Reverter D. (2020). Molecular mechanisms in SUMO conjugation. Biochem. Soc. Trans..

[31] Kunadis E., Lakiotaki E., Korkolopoulou P., Piperi C. (2021). Targeting post-translational histone modifying enzymes in glioblastoma. Pharmacol. Ther..

[32] Lara-Urena N., Jafari V., Garcia-Dominguez M. (2022). Cancer-Associated Dysregulation of Sumo Regulators: Proteases and Ligases. Int. J. Mol. Sci..

[33] Yang X., Khosravi-Far R., Chang H.Y., Baltimore D. (1997). Daxx, a novel Fas-binding protein that activates JNK and apoptosis. Cell.

[34] Chang H.Y., Nishitoh H., Yang X., Ichijo H., Baltimore D. (1998). Activation of apoptosis signal-regulating kinase 1 (ASK1) by the adapter protein Daxx. Science.

[35] Santiago A., Godsey A.C., Hossain J., Zhao L.Y., Liao D. (2009). Identification of two independent SUMO-interacting motifs in Daxx: Evolutionary conservation from Drosophila to humans and their biochemical functions. Cell Cycle.

[36] Michaelson J.S., Bader D., Kuo F., Kozak C., Leder P. (1999). Loss of Daxx, a promiscuously interacting protein, results in extensive apoptosis in early mouse development. Genes Dev..

[37] Wasylishen A.R., Estrella J.S., Pant V., Chau G.P., Lozano G. (2018). Daxx Functions Are p53-Independent In Vivo. Mol. Cancer Res..

[38] Escobar-Cabrera E., Lau D.K., Giovinazzi S., Ishov A.M., McIntosh L.P. (2010). Structural characterization of the DAXX N-terminal helical bundle domain and its complex with Rassf1C. Structure.

[39] Mahmud I., Liao D. (2019). DAXX in cancer: Phenomena, processes, mechanisms and regulation. Nucleic Acids Res..

[40] Hollenbach A.D., Sublett J.E., McPherson C.J., Grosveld G. (1999). The Pax3-FKHR oncoprotein is unresponsive to the Pax3-associated repressor hDaxx. EMBO J..

[41] Ishov A.M., Sotnikov A.G., Negorev D., Vladimirova O.V., Neff N., Kamitani T., Yeh E.T., Strauss J.F.R., Maul G.G. (1999). PML is critical for ND10 formation and recruits the PML-interacting protein daxx to this nuclear structure when modified by SUMO-1. J. Cell Biol..

[42] Li H., Leo C., Zhu J., Wu X., O’Neil J., Park E.J., Chen J.D. (2000). Sequestration and inhibition of Daxx-mediated transcriptional repression by PML. Mol. Cell. Biol..

[43] Lehembre F., Müller S., Pandolfi P.P., Dejean A. (2001). Regulation of Pax3 transcriptional activity by SUMO-1-modified PML. Oncogene.

[44] Hollenbach A.D., McPherson C.J., Mientjes E.J., Iyengar R., Grosveld G. (2002). Daxx and histone deacetylase II associate with chromatin through an interaction with core histones and the chromatin-associated protein Dek. J. Cell Sci..

[45] Lin D.Y., Huang Y.S., Jeng J.C., Kuo H.Y., Chang C.C., Chao T.T., Ho C.C., Chen Y.C., Lin T.P., Fang H.I. (2006). Role of SUMO-interacting motif in Daxx SUMO modification, subnuclear localization, and repression of sumoylated transcription factors. Mol. Cell.

[46] Lewis P.W., Elsaesser S.J., Noh K.M., Stadler S.C., Allis C.D. (2010). Daxx is an H3.3-specific histone chaperone and cooperates with ATRX in replication-independent chromatin assembly at telomeres. Proc. Natl. Acad. Sci. USA.

[47] Drane P., Ouararhni K., Depaux A., Shuaib M., Hamiche A. (2010). The death-associated protein DAXX is a novel histone chaperone involved in the replication-independent deposition of H3.3. Genes Dev..

[48] Falcinelli M., Dell’Omo G., Grassi E., Mariella E., Leto S.M., Scardellato S., Lorenzato A., Arena S., Bertotti A., Trusolino L. (2023). Colorectal cancer patient-derived organoids and cell lines harboring *ATRX* and/or *DAXX* mutations lack Alternative Lengthening of Telomeres (ALT). Cell Death Dis..

[49] Lovejoy C.A., Li W., Reisenweber S., Thongthip S., Bruno J., de Lange T., De S., Petrini J.H., Sung P.A., Jasin M. (2012). Loss of ATRX, genome instability, and an altered DNA damage response are hallmarks of the alternative lengthening of telomeres pathway. PLoS Genet..

[50] Huang L., Agrawal T., Zhu G., Yu S., Tao L., Lin J., Marmorstein R., Shorter J., Yang X. (2021). DAXX represents a new type of protein-folding enabler. Nature.

[51] Chang C.C., Naik M.T., Huang Y.S., Jeng J.C., Liao P.H., Kuo H.Y., Ho C.C., Hsieh Y.L., Lin C.H., Huang N.J. (2011). Structural and functional roles of Daxx SIM phosphorylation in SUMO paralog-selective binding and apoptosis modulation. Mol. Cell.

[52] Li L., Wen J., Tuo Q.H., Liao D.F. (2013). [Effects of SUMOylation on the subcellular localization and function of DAXX]. Sheng Li Xue Bao.

[53] Escobar-Cabrera E., Okon M., Lau D.K., Dart C.F., Bonvin A.M., McIntosh L.P. (2011). Characterizing the N- and C-terminal Small ubiquitin-like modifier (SUMO)-interacting motifs of the scaffold protein DAXX. J. Biol. Chem..

[54] Hecker C.M., Rabiller M., Haglund K., Bayer P., Dikic I. (2006). Specification of SUMO1- and SUMO2-interacting motifs. J. Biol. Chem..

[55] Stehmeier P., Muller S. (2009). Phospho-regulated SUMO interaction modules connect the SUMO system to CK2 signaling. Mol. Cell.

[56] Cappadocia L., Pichler A., Lima C.D. (2015). Structural basis for catalytic activation by the human ZNF451 SUMO E3 ligase. Nat. Struct. Mol. Biol..

[57] Ishov A.M., Vladimirova O.V., Maul G.G. (2002). Daxx-mediated accumulation of human cytomegalovirus tegument protein pp71 at ND10 facilitates initiation of viral infection at these nuclear domains. J. Virol..

[58] Lalioti V.S., Vergarajauregui S., Pulido D., Sandoval I.V. (2002). The insulin-sensitive glucose transporter, GLUT_4_, interacts physically with Daxx. Two proteins with capacity to bind Ubc_9_ and conjugated to SUMO1. J. Biol. Chem..

[59] Lalioti V.S., Vergarajauregui S., Tsuchiya Y., Hernandez-Tiedra S., Sandoval I.V. (2009). Daxx functions as a scaffold of a protein assembly constituted by GLUT4, JNK1 and KIF5B. J. Cell. Physiol..

[60] Rubin B.R., Bogan J.S. (2009). Intracellular retention and insulin-stimulated mobilization of GLUT4 glucose transporters. Vitam. Horm..

[61] Hu J., Wang S., Xiong Z., Cheng Z., Yang Z., Lin J., Wang T., Feng X., Gao E., Wang H. (2018). Exosomal Mst1 transfer from cardiac microvascular endothelial cells to cardiomyocytes deteriorates diabetic cardiomyopathy. Biochim. Biophys. Acta Mol. Basis Dis..

[62] Li R., Pei H., Watson D.K., Papas T.S. (2000). EAP1/Daxx interacts with ETS1 and represses transcriptional activation of ETS1 target genes. Oncogene.

[63] Mahmud I., Tian G., Wang J., Hutchinson T.E., Kim B.J., Awasthee N., Hale S., Meng C., Moore A., Zhao L. (2023). DAXX drives de novo lipogenesis and contributes to tumorigenesis. Nat. Commun..

[64] Gurer C., Berthoux L., Luban J. (2005). Covalent modification of human immunodeficiency virus type 1 p6 by SUMO-1. J. Virol..

[65] Li X.D., Makela T.P., Guo D., Soliymani R., Koistinen V., Vapalahti O., Vaheri A., Lankinen H. (2002). Hantavirus nucleocapsid protein interacts with the Fas-mediated apoptosis enhancer Daxx. J. Gen. Virol..

[66] Kuo H.Y., Chang C.C., Jeng J.C., Hu H.M., Lin D.Y., Maul G.G., Kwok R.P., Shih H.M. (2005). SUMO modification negatively modulates the transcriptional activity of CREB-binding protein via the recruitment of Daxx. Proc. Natl. Acad. Sci. USA.

[67] Geiss-Friedlander R., Melchior F. (2007). Concepts in sumoylation: A decade on. Nat. Rev. Mol. Cell Biol..

[68] Spector D.L. (2001). Nuclear domains. J. Cell Sci..

[69] Wang I.F., Reddy N.M., Shen C.K. (2002). Higher order arrangement of the eukaryotic nuclear bodies. Proc. Natl. Acad. Sci. USA.

[70] Lang M., Jegou T., Chung I., Richter K., Munch S., Udvarhelyi A., Cremer C., Hemmerich P., Engelhardt J., Hell S.W. (2010). Three-dimensional organization of promyelocytic leukemia nuclear bodies. J. Cell Sci..

[71] Kleijwegt C., Bressac F., Seurre C., Bouchereau W., Cohen C., Texier P., Simonet T., Schaeffer L., Lomonte P., Corpet A. (2023). Interplay between PML NBs and HIRA for H3.3 dynamics following type I interferon stimulus. eLife.

[72] Cheng X., Kao H.Y. (2012). Post-translational modifications of PML: Consequences and implications. Front. Oncol..

[73] Boddy M.N., Howe K., Etkin L.D., Solomon E., Freemont P.S. (1996). PIC 1, a novel ubiquitin-like protein which interacts with the PML component of a multiprotein complex that is disrupted in acute promyelocytic leukaemia. Oncogene.

[74] Muller S., Matunis M.J., Dejean A. (1998). Conjugation with the ubiquitin-related modifier SUMO-1 regulates the partitioning of PML within the nucleus. EMBO J..

[75] Zhong S., Müller S., Ronchetti S., Freemont P.S., Dejean A., Pandolfi P.P. (2000). Role of SUMO-1-modified PML in nuclear body formation. Blood.

[76] Geng Y., Monajembashi S., Shao A., Cui D., He W., Chen Z., Hemmerich P., Tang J. (2012). Contribution of the C-terminal regions of promyelocytic leukemia protein (PML) isoforms II and V to PML nuclear body formation. J. Biol. Chem..

[77] Shen T.H., Lin H.K., Scaglioni P.P., Yung T.M., Pandolfi P.P. (2006). The mechanisms of PML-nuclear body formation. Mol. Cell.

[78] Gao C., Ho C.C., Reineke E., Lam M., Cheng X., Stanya K.J., Liu Y., Chakraborty S., Shih H.M., Kao H.Y. (2008). Histone deacetylase 7 promotes PML sumoylation and is essential for PML nuclear body formation. Mol. Cell. Biol..

[79] Cappadocia L., Mascle X.H., Bourdeau V., Tremblay-Belzile S., Chaker-Margot M., Lussier-Price M., Wada J., Sakaguchi K., Aubry M., Ferbeyre G. (2015). Structural and functional characterization of the phosphorylation-dependent interaction between PML and SUMO1. Structure.

[80] Matunis M.J., Zhang X.D., Ellis N.A. (2006). SUMO: The glue that binds. Dev. Cell.

[81] Jeanne M., Lallemand-Breitenbach V., Ferhi O., Koken M., Le Bras M., Duffort S., Peres L., Berthier C., Soilihi H., Raught B. (2010). PML/RARA oxidation and arsenic binding initiate the antileukemia response of As_2_O_3_. Cancer Cell.

[82] Sahin U., Ferhi O., Jeanne M., Benhenda S., Berthier C., Jollivet F., Niwa-Kawakita M., Faklaris O., Setterblad N., de The H. (2014). Oxidative stress-induced assembly of PML nuclear bodies controls sumoylation of partner proteins. J. Cell Biol..

[83] Wang P., Benhenda S., Wu H., Lallemand-Breitenbach V., Zhen T., Jollivet F., Peres L., Li Y., Chen S.J., Chen Z. (2018). RING tetramerization is required for nuclear body biogenesis and PML sumoylation. Nat. Commun..

[84] Li Y., Ma X., Chen Z., Wu H., Wang P., Wu W., Cheng N., Zeng L., Zhang H., Cai X. (2019). B1 oligomerization regulates PML nuclear body biogenesis and leukemogenesis. Nat. Commun..

[85] Ching R.W., Dellaire G., Eskiw C.H., Bazett-Jones D.P. (2005). PML bodies: A meeting place for genomic loci?. J. Cell Sci..

[86] El B.J., Dianoux L., Chelbi-Alix M.K. (2011). PML positively regulates interferon gamma signaling. Biochimie.

[87] Ulbricht T., Alzrigat M., Horch A., Reuter N., von Mikecz A., Steimle V., Schmitt E., Kramer O.H., Stamminger T., Hemmerich P. (2012). PML promotes MHC class II gene expression by stabilizing the class II transactivator. J. Cell Biol..

[88] Chen Y., Wright J., Meng X., Leppard K.N. (2015). Promyelocytic Leukemia Protein Isoform II Promotes Transcription Factor Recruitment To Activate Interferon Beta and Interferon-Responsive Gene Expression. Mol. Cell. Biol..

[89] Dellaire G., Bazett-Jones D.P. (2004). PML nuclear bodies: Dynamic sensors of DNA damage and cellular stress. Bioessays.

[90] Bernardi R., Pandolfi P.P. (2007). Structure, dynamics and functions of promyelocytic leukaemia nuclear bodies. Nat. Rev. Mol. Cell Biol..

[91] Chang H.R., Munkhjargal A., Kim M.J., Park S.Y., Jung E., Ryu J.H., Yang Y., Lim J.S., Kim Y. (2018). The functional roles of PML nuclear bodies in genome maintenance. Mutat. Res..

[92] Corpet A., Kleijwegt C., Roubille S., Juillard F., Jacquet K., Texier P., Lomonte P. (2020). PML nuclear bodies and chromatin dynamics: Catch me if you can!. Nucleic Acids Res..

[93] Spegg V., Altmeyer M. (2024). Genome maintenance meets mechanobiology. Chromosoma.

[94] Chung I., Osterwald S., Deeg K.I., Rippe K. (2012). PML body meets telomere: The beginning of an ALTernate ending?. Nucleus.

[95] Ryabchenko B., Sroller V., Hornikova L., Lovtsov A., Forstova J., Huerfano S. (2023). The interactions between PML nuclear bodies and small and medium size DNA viruses. Virol. J..

[96] Pichler A., Fatouros C., Lee H., Eisenhardt N. (2017). SUMO conjugation—A mechanistic view. Biomol. Concepts.

[97] Dellaire G., Bazett-Jones D.P. (2007). Beyond repair foci: Subnuclear domains and the cellular response to DNA damage. Cell Cycle.

[98] Palvimo J.J. (2007). PIAS proteins as regulators of small ubiquitin-related modifier (SUMO) modifications and transcription. Biochem. Soc. Trans..

[99] Schmidt D., Muller S. (2003). PIAS/SUMO: New partners in transcriptional regulation. Cell. Mol. Life Sci..

[100] Ullmann R., Chien C.D., Avantaggiati M.L., Muller S. (2012). An acetylation switch regulates SUMO-dependent protein interaction networks. Mol. Cell.

[101] Deribe Y.L., Pawson T., Dikic I. (2010). Post-translational modifications in signal integration. Nat. Struct. Mol. Biol..

[102] Scaglioni P.P., Yung T.M., Cai L.F., Erdjument-Bromage H., Kaufman A.J., Singh B., Teruya-Feldstein J., Tempst P., Pandolfi P.P. (2006). A CK2-dependent mechanism for degradation of the PML tumor suppressor. Cell.

[103] Rabellino A., Carter B., Konstantinidou G., Wu S.Y., Rimessi A., Byers L.A., Heymach J.V., Girard L., Chiang C.M., Teruya-Feldstein J. (2012). The SUMO E3-ligase PIAS1 regulates the tumor suppressor PML and its oncogenic counterpart PML-RARA. Cancer Res..

[104] Cho G., Lim Y., Golden J.A. (2009). SUMO interaction motifs in Sizn1 are required for promyelocytic leukemia protein nuclear body localization and for transcriptional activation. J. Biol. Chem..

[105] Negorev D., Ishov A.M., Maul G.G. (2001). Evidence for separate ND10-binding and homo-oligomerization domains of Sp100. J. Cell Sci..

[106] Rasheed Z.A., Saleem A., Ravee Y., Pandolfi P.P., Rubin E.H. (2002). The topoisomerase I-binding RING protein, topors, is associated with promyelocytic leukemia nuclear bodies. Exp. Cell Res..

[107] Sung K.S., Lee Y.A., Kim E.T., Lee S.R., Ahn J.H., Choi C.Y. (2011). Role of the SUMO-interacting motif in HIPK2 targeting to the PML nuclear bodies and regulation of p53. Exp. Cell. Res..

[108] Mukhopadhyay D., Matunis M.J. (2011). SUMmOning Daxx-mediated repression. Mol. Cell.

[109] Cheema A., Knights C.D., Rao M., Catania J., Perez R., Simons B., Dakshanamurthy S., Kolukula V.K., Tilli M., Furth P.A. (2010). Functional mimicry of the acetylated C-terminal tail of p53 by a SUMO-1 acetylated domain, SAD. J. Cell. Physiol..

[110] Mascle X.H., Gagnon C., Wahba H.M., Lussier-Price M., Cappadocia L., Sakaguchi K., Omichinski J.G. (2020). Acetylation of SUMO1 Alters Interactions with the SIMs of PML and Daxx in a Protein-Specific Manner. Structure.

[111] Ji Z., Degerny C., Vintonenko N., Deheuninck J., Foveau B., Leroy C., Coll J., Tulasne D., Baert J.L., Fafeur V. (2007). Regulation of the Ets-1 transcription factor by sumoylation and ubiquitinylation. Oncogene.

[112] Hua G., Ganti K.P., Chambon P. (2016). Glucocorticoid-induced tethered transrepression requires SUMOylation of GR and formation of a SUMO-SMRT/NCoR1-HDAC3 repressing complex. Proc. Natl. Acad. Sci. USA.

[113] Lin D.Y., Lai M.Z., Ann D.K., Shih H.M. (2003). Promyelocytic leukemia protein (PML) functions as a glucocorticoid receptor co-activator by sequestering Daxx to the PML oncogenic domains (PODs) to enhance its transactivation potential. J. Biol. Chem..

[114] Lee P.S., Chang C., Liu D., Derynck R. (2003). Sumoylation of Smad_4_, the common Smad mediator of transforming growth factor-β family signaling. J. Biol. Chem..

[115] Chang C.C., Lin D.Y., Fang H.I., Chen R.H., Shih H.M. (2005). Daxx mediates the small ubiquitin-like modifier-dependent transcriptional repression of Smad_4_. J. Biol. Chem..

[116] Lin D.Y., Fang H.I., Ma A.H., Huang Y.S., Pu Y.S., Jenster G., Kung H.J., Shih H.M. (2004). Negative modulation of androgen receptor transcriptional activity by Daxx. Mol. Cell. Biol..

[117] Poukka H., Karvonen U., Janne O.A., Palvimo J.J. (2000). Covalent modification of the androgen receptor by small ubiquitin-like modifier 1 (SUMO-1). Proc. Natl. Acad. Sci. USA.

[118] Meloni A., Fiorillo E., Corda D., Incani F., Serra M.L., Contini A., Cao A., Rosatelli M.C. (2010). DAXX is a new AIRE-interacting protein. J. Biol. Chem..

[119] Lin C.W., Wang L.K., Wang S.P., Chang Y.L., Wu Y.Y., Chen H.Y., Hsiao T.H., Lai W.Y., Lu H.H., Chang Y.H. (2016). Daxx inhibits hypoxia-induced lung cancer cell metastasis by suppressing the HIF-1alpha/HDAC1/Slug axis. Nat. Commun..

[120] Xie Y., Liu S., Lu W., Yang Q., Williams K.D., Binhazim A.A., Carver B.S., Matusik R.J., Chen Z. (2014). Slug regulates E-cadherin repression via p19Arf in prostate tumorigenesis. Mol. Oncol..

[121] Yamamoto H., Ihara M., Matsuura Y., Kikuchi A. (2003). Sumoylation is involved in β-catenin-dependent activation of Tcf-4. EMBO J..

[122] Tzeng S.L., Cheng Y.W., Li C.H., Lin Y.S., Hsu H.C., Kang J.J. (2006). Physiological and functional interactions between Tcf_4_ and Daxx in colon cancer cells. J. Biol. Chem..

[123] Zhou Z., Wang M., Li J., Xiao M., Chin Y.E., Cheng J., Yeh E.T., Yang J., Yi J. (2016). SUMOylation and SENP3 regulate STAT3 activation in head and neck cancer. Oncogene.

[124] Muromoto R., Nakao K., Watanabe T., Sato N., Sekine Y., Sugiyama K., Oritani K., Shimoda K., Matsuda T. (2006). Physical and functional interactions between Daxx and STAT3. Oncogene.

[125] Feng Z.J., Gurung B., Jin G.H., Yang X.L., Hua X.X. (2013). SUMO modification of menin. Am. J. Cancer Res..

[126] Feng Z., Wang L., Sun Y., Jiang Z., Domsic J., An C., Xing B., Tian J., Liu X., Metz D.C. (2017). Menin and Daxx Interact to Suppress Neuroendocrine Tumors through Epigenetic Control of the Membrane Metallo-Endopeptidase. Cancer Res..

[127] Liu Y., Bridges R., Wortham A., Kulesz-Martin M. (2012). NF-kappaB repression by PIAS3 mediated RelA SUMOylation. PLoS ONE.

[128] Maroulakou I.G., Bowe D.B. (2000). Expression and function of Ets transcription factors in mammalian development: A regulatory network. Oncogene.

[129] Zhang C., Kavurma M.M., Lai A., Khachigian L.M. (2003). Ets-1 protects vascular smooth muscle cells from undergoing apoptosis by activating p21^WAF1/Cip1^: ETS-1 regulates basal and and inducible p21^WAF1/Cip1^: ETS-1 regulates basal and inducible p21^WAF1/Cip1^ transcription via distinct cis-acting elements in the p21^WAF/Cip1^ promoter. J. Biol. Chem..

[130] Marziali G., Perrotti E., Ilari R., Lulli V., Coccia E.M., Moret R., Kuhn L.C., Testa U., Battistini A. (2002). Role of Ets-1 in transcriptional regulation of transferrin receptor and erythroid differentiation. Oncogene.

[131] Naito S., Shimizu S., Matsuu M., Nakashima M., Nakayama T., Yamashita S., Sekine I. (2002). Ets-1 upregulates matrix metalloproteinase-1 expression through extracellular matrix adhesion in vascular endothelial cells. Biochem. Biophys. Res. Commun..

[132] Iwasaka C., Tanaka K., Abe M., Sato Y. (1996). Ets-1 regulates angiogenesis by inducing the expression of urokinase-type plasminogen activator and matrix metalloproteinase-1 and the migration of vascular endothelial cells. J. Cell. Physiol..

[133] Chen Z., Fisher R.J., Riggs C.W., Rhim J.S., Lautenberger J.A. (1997). Inhibition of vascular endothelial growth factor-induced endothelial cell migration by ETS1 antisense oligonucleotides. Cancer Res..

[134] Oda N., Abe M., Sato Y. (1999). ETS-1 converts endothelial cells to the angiogenic phenotype by inducing the expression of matrix metalloproteinases and integrin β_3_. J. Cell. Physiol..

[135] Xu D., Wilson T.J., Chan D., De Luca E., Zhou J., Hertzog P.J., Kola I. (2002). Ets1 is required for p53 transcriptional activity in UV-induced apoptosis in embryonic stem cells. EMBO J..

[136] Timmermans S., Souffriau J., Libert C. (2019). A General Introduction to Glucocorticoid Biology. Front. Immunol..

[137] Cruz-Topete D., Cidlowski J.A. (2015). One hormone, two actions: Anti- and pro-inflammatory effects of glucocorticoids. Neuroimmunomodulation.

[138] Wang J.C., Gray N.E., Kuo T., Harris C.A. (2012). Regulation of triglyceride metabolism by glucocorticoid receptor. Cell Biosci..

[139] Tassabehji M., Read A.P., Newton V.E., Harris R., Balling R., Gruss P., Strachan T. (1992). Waardenburg’s syndrome patients have mutations in the human homologue of the Pax-3 paired box gene. Nature.

[140] Qi J., Yan L., Sun J., Huang C., Su B., Cheng J., Shen L. (2024). SUMO-specific protease 1 regulates germinal center B cell response through deSUMOylation of PAX5. Proc. Natl. Acad. Sci. USA.

[141] Yan Q., Gong L., Deng M., Zhang L., Sun S., Liu J., Ma H., Yuan D., Chen P.C., Hu X. (2010). Sumoylation activates the transcriptional activity of Pax-6, an important transcription factor for eye and brain development. Proc. Natl. Acad. Sci. USA.

[142] Luan Z., Liu Y., Stuhlmiller T.J., Marquez J., Garcia-Castro M.I. (2013). SUMOylation of Pax7 is essential for neural crest and muscle development. Cell. Mol. Life Sci..

[143] Emelyanov A.V., Kovac C.R., Sepulveda M.A., Birshtein B.K. (2002). The interaction of Pax_5_ (BSAP) with Daxx can result in transcriptional activation in B cells. J. Biol. Chem..

[144] Igalouzene R., Hernandez-Vargas H., Benech N., Guyennon A., Bauche D., Barrachina C., Dubois E., Marie J.C., Soudja S.M. (2022). SMAD4 TGF-β-independent function preconditions naive CD8^+^ T cells to prevent severe chronic intestinal inflammation. J. Clin. Invest..

[145] Li T.P., Sun S.W., Xiong G.Z., Qiu F., Yang D.M., Sun S.Y., Xie X.J., Liao D.F., Chen J.X., Tuo Q.H. (2021). Direct Interaction of Daxx and Androgen Receptor Is Required for Their Regulatory Activity in Cholesterol Biosynthesis. Pharmacology.

[146] Sun S., Wen J., Qiu F., Yin Y., Xu G., Li T., Nie J., Xiong G., Zhang C., Liao D. (2016). Identification of the C-terminal domain of Daxx acts as a potential regulator of intracellular cholesterol synthesis in HepG2 cells. Biochem. Biophys. Res. Commun..

[147] Anderson M.S., Venanzi E.S., Klein L., Chen Z., Berzins S.P., Turley S.J., von Boehmer H., Bronson R., Dierich A., Benoist C. (2002). Projection of an immunological self shadow within the thymus by the aire protein. Science.

[148] Sansom S.N., Shikama-Dorn N., Zhanybekova S., Nusspaumer G., Macaulay I.C., Deadman M.E., Heger A., Ponting C.P., Hollander G.A. (2014). Population and single-cell genomics reveal the Aire dependency, relief from Polycomb silencing, and distribution of self-antigen expression in thymic epithelia. Genome Res..

[149] Meredith M., Zemmour D., Mathis D., Benoist C. (2015). Aire controls gene expression in the thymic epithelium with ordered stochasticity. Nat. Immunol..

[150] Barrallo-Gimeno A., Nieto M.A. (2005). The Snail genes as inducers of cell movement and survival: Implications in development and cancer. Development.

[151] Nieto M.A. (2002). The snail superfamily of zinc-finger transcription factors. Nat. Rev. Mol. Cell Biol..

[152] Hung P.F., Hong T.M., Chang C.C., Hung C.L., Hsu Y.L., Chang Y.L., Wu C.T., Chang G.C., Chan N.L., Yu S.L. (2019). Hypoxia-induced Slug SUMOylation enhances lung cancer metastasis. J. Exp. Clin. Cancer Res..

[153] Obradovic D., Tirard M., Nemethy Z., Hirsch O., Gronemeyer H., Almeida O.F. (2004). DAXX, FLASH, and FAF-1 modulate mineralocorticoid and glucocorticoid receptor-mediated transcription in hippocampal cells—Toward a basis for the opposite actions elicited by two nuclear receptors?. Mol. Pharmacol..

[154] Muromoto R., Kuroda M., Togi S., Sekine Y., Nanbo A., Shimoda K., Oritani K., Matsuda T. (2010). Functional involvement of Daxx in gp130-mediated cell growth and survival in BaF3 cells. Eur. J. Immunol..

[155] Chao W., Shen Y., Li L., Zhao H., Meiler S.E., Cook S.A., Rosenzweig A. (2005). Fas-associated death-domain protein inhibits TNF-alpha mediated NF-kappaB activation in cardiomyocytes. Am. J. Physiol. Heart Circ. Physiol..

[156] Park J., Lee J.H., La M., Jang M.J., Chae G.W., Kim S.B., Tak H., Jung Y., Byun B., Ahn J.K. (2007). Inhibition of NF-kappaB acetylation and its transcriptional activity by Daxx. J. Mol. Biol..

[157] Puto L.A., Reed J.C. (2008). Daxx represses RelB target promoters via DNA methyltransferase recruitment and DNA hypermethylation. Genes Dev..

[158] Zhao Y.Q., Jin H.R., Kim D., Jung S.H., Liu S., Wan J., Lo H.Y., Fu X.Q., Wang Q., Hao C. (2023). SUMO1 degrader induces ER stress and ROS accumulation through deSUMOylation of TCF4 and inhibition of its transcription of StarD7 in colon cancer. Mol. Carcinog..

[159] Ihara M., Yamamoto H., Kikuchi A. (2005). SUMO-1 modification of PIASy, an E3 ligase, is necessary for PIASy-dependent activation of Tcf-4. Mol. Cell. Biol..

[160] Liu H., Zhang J., Xue Z., Chang M., Feng X., Cai Y., Bai L., Wang W., Liu E., Zhao S. (2023). Deficiency of protein inhibitor of activated STAT3 exacerbates atherosclerosis by modulating VSMC phenotypic switching. Atherosclerosis.

[161] Nayak A., Viale-Bouroncle S., Morsczeck C., Muller S. (2014). The SUMO-specific isopeptidase SENP3 regulates MLL1/MLL2 methyltransferase complexes and controls osteogenic differentiation. Mol. Cell.

[162] Liu J., Wu Z., Han D., Wei C., Liang Y., Jiang T., Chen L., Sha M., Cao Y., Huang F. (2020). Mesencephalic Astrocyte-Derived Neurotrophic Factor Inhibits Liver Cancer Through Small Ubiquitin-Related Modifier (SUMO)ylation-Related Suppression of NF-kappaB/Snail Signaling Pathway and Epithelial-Mesenchymal Transition. Hepatology.

[163] Kracklauer M.P., Schmidt C. (2003). At the crossroads of SUMO and NF-kappaB. Mol. Cancer.

[164] Chen S., Fu X., Wang R., Li M., Yan X., Yue Z., Chen S.W., Dong M., Xu A., Huang S. (2023). SUMO and PIAS repress NF-kappaB activation in a basal chordate. Fish Shellfish Immunol..

[165] Wang C.H., Hung P.W., Chiang C.W., Lombes M., Chen C.H., Lee K.H., Lo Y.C., Wu M.H., Chang W.C., Lin D.Y. (2019). Identification of two independent SUMO-interacting motifs in Fas-associated factor 1 (FAF1): Implications for mineralocorticoid receptor (MR)-mediated transcriptional regulation. Biochim. Biophys. Acta Mol. Cell Res..

[166] Wu C., Ding H., Wang S., Li Y., Liu S.B., Wang X., Zheng J., Xue T., Amin H.M., Song Y.H. (2020). DAXX inhibits cancer stemness and epithelial-mesenchymal transition in gastric cancer. Br. J. Cancer.

[167] Peiffer D.S., Wyatt D., Zlobin A., Piracha A., Ng J., Dingwall A.K., Albain K.S., Osipo C. (2019). DAXX Suppresses Tumor-Initiating Cells in Estrogen Receptor-Positive Breast Cancer Following Endocrine Therapy. Cancer Res..

[168] Muromoto R., Sugiyama K., Takachi A., Imoto S., Sato N., Yamamoto T., Oritani K., Shimoda K., Matsuda T. (2004). Physical and functional interactions between Daxx and DNA methyltransferase 1-associated protein, DMAP1. J. Immunol..

[169] Ecsedy J.A., Michaelson J.S., Leder P. (2003). Homeodomain-interacting protein kinase 1 modulates Daxx localization, phosphorylation, and transcriptional activity. Mol. Cell. Biol..

[170] Bogolyubova I.O., Sailau Z.K., Bogolyubov D.S. (2019). The dynamics of DAXX protein distribution in the nucleus of mouse early embryos. Acta Histochem..

[171] Muromoto R. (2012). [Death domain-associated protein (DAXX)-mediated regulation of transcription and cell death]. Yakugaku Zasshi.

[172] Meinecke I., Cinski A., Baier A., Peters M.A., Dankbar B., Wille A., Drynda A., Mendoza H., Gay R.E., Hay R.T. (2007). Modification of nuclear PML protein by SUMO-1 regulates Fas-induced apoptosis in rheumatoid arthritis synovial fibroblasts. Proc. Natl. Acad. Sci. USA.

[173] Song J.J., Lee Y.J. (2004). Tryptophan 621 and serine 667 residues of Daxx regulate its nuclear export during glucose deprivation. J. Biol. Chem..

[174] Junn E., Taniguchi H., Jeong B.S., Zhao X., Ichijo H., Mouradian M.M. (2005). Interaction of DJ-1 with Daxx inhibits apoptosis signal-regulating kinase 1 activity and cell death. Proc. Natl. Acad. Sci. USA.

[175] Saeed U., Ray A., Valli R.K., Kumar A.M., Ravindranath V. (2010). DJ-1 loss by glutaredoxin but not glutathione depletion triggers Daxx translocation and cell death. Antioxid. Redox Signal..

[176] Lind-Holm Mogensen F., Sousa C., Ameli C., Badanjak K., Pereira S.L., Muller A., Kaoma T., Coowar D., Scafidi A., Poovathingal S.K. (2024). *PARK7*/DJ-1 deficiency impairs microglial activation in response to LPS-induced inflammation. J. Neuroinflammation.

[177] Guo T., Zhou L., Xiong M., Xiong J., Huang J., Li Y., Zhang G., Chen G., Wang Z.H., Xiao T. (2024). N-homocysteinylation of DJ-1 promotes neurodegeneration in Parkinson’s disease. Aging Cell.

[178] Karunakaran S., Diwakar L., Saeed U., Agarwal V., Ramakrishnan S., Iyengar S., Ravindranath V. (2007). Activation of apoptosis signal regulating kinase 1 (ASK1) and translocation of death-associated protein, Daxx, in substantia nigra pars compacta in a mouse model of Parkinson’s disease: Protection by alpha-lipoic acid. FASEB J..

[179] Hwang S., Song S., Hong Y.K., Choi G., Suh Y.S., Han S.Y., Lee M., Park S.H., Lee J.H., Lee S. (2013). *Drosophila* DJ-1 decreases neural sensitivity to stress by negatively regulating Daxx-like protein through dFOXO. PLoS Genet..

[180] Grimwade D., Lo Coco F. (2002). Acute promyelocytic leukemia: A model for the role of molecular diagnosis and residual disease monitoring in directing treatment approach in acute myeloid leukemia. Leukemia.

[181] Nowak D., Stewart D., Koeffler H.P. (2009). Differentiation therapy of leukemia: 3 decades of development. Blood.

[182] Sirulnik A., Melnick A., Zelent A., Licht J.D. (2003). Molecular pathogenesis of acute promyelocytic leukaemia and APL variants. Best. Pract. Res. Clin. Haematol..

[183] Salsman J., Rapkin L.M., Margam N.N., Duncan R., Bazett-Jones D.P., Dellaire G. (2017). Myogenic differentiation triggers PML nuclear body loss and DAXX relocalization to chromocentres. Cell Death Dis..

[184] Zhong S., Salomoni P., Ronchetti S., Guo A., Ruggero D., Pandolfi P.P. (2000). Promyelocytic leukemia protein (PML) and Daxx participate in a novel nuclear pathway for apoptosis. J. Exp. Med..

[185] Torii S., Egan D.A., Evans R.A., Reed J.C. (1999). Human Daxx regulates Fas-induced apoptosis from nuclear PML oncogenic domains (PODs). EMBO J..

[186] Wethkamp N., Klempnauer K.H. (2009). Daxx is a transcriptional repressor of CCAAT/enhancer-binding protein β. J. Biol. Chem..

[187] Duprez E., Wagner K., Koch H., Tenen D.G. (2003). C/EBPβ: A major PML-RARA-responsive gene in retinoic acid-induced differentiation of APL cells. EMBO J..

[188] Lin R.J., Egan D.A., Evans R.M. (1999). Molecular genetics of acute promyelocytic leukemia. Trends Genet..

[189] Sung H., Ferlay J., Siegel R.L., Laversanne M., Soerjomataram I., Jemal A., Bray F. (2021). Global Cancer Statistics 2020: GLOBOCAN Estimates of Incidence and Mortality Worldwide for 36 Cancers in 185 Countries. CA Cancer J. Clin..

[190] Joshi S.S., Badgwell B.D. (2021). Current treatment and recent progress in gastric cancer. CA Cancer J. Clin..

[191] Xu J.F., Zhao Z.G., Ye L.L., Zhuge W., Han Z., Zhang T.M., Ye S.S., Chen W.J., Zhu S., Shi L. (2017). Prognostic significance of Daxx NCR (Nuclear/Cytoplasmic Ratio) in gastric cancer. Cancer Med..

[192] Chen C., Sun X., Xie W., Chen S., Hu Y., Xing D., Xu J., Chen X., Zhao Z., Han Z. (2020). Opposing biological functions of the cytoplasm and nucleus DAXX modified by SUMO-2/3 in gastric cancer. Cell Death Dis..

[193] Huang Y.S., Shih H.M. (2009). Daxx positively modulates β-catenin/TCF4-mediated transcriptional potential. Biochem. Biophys. Res. Commun..

